# Depriving Out-of-School Children of Deworming Tablets for Soil-Transmitted Helminth Infection in Bangladesh: The Irony of a School-Based Deworming Programme

**DOI:** 10.3390/tropicalmed7030035

**Published:** 2022-02-24

**Authors:** Avijit Saha, Srizan Chowdhury, Edwin Theophilus Goswami, Konica Gop, Ariful Alam, Asadur Rahman, Malabika Sarker

**Affiliations:** 1 BRAC James P Grant School of Public Health, BRAC University, Dhaka 1213, Bangladesh; srizan.chowdhury@icddrb.org (S.C.); malabika@bracu.ac.bd (M.S.); 2 HNPP, BRAC, BRAC Centre, 75 Mohakhali, Dhaka 1212, Bangladesh; egoswami@nutritionintl.org (E.T.G.); konica.gop@brac.net (K.G.); ariful.a@brac.net (A.A.); asadur.rahman@brac.net (A.R.); 3 Heidelberg Institute of Global Health, Heidelberg University, Im Neuenheimer Feld 130/3, 69120 Heidelberg, Germany

**Keywords:** deworming, SACs, OSCs, SGCs, school-based deworming, coverage

## Abstract

Since 2008, Bangladesh has had a school-based deworming programme to combat soil-transmitted helminth (STH) infection among school-aged children (SACs). Existing programmes have trouble reaching SACs, especially those out-of-school (OSCs). This study evaluated deworming coverage among school going children (SGCs) and OSCs in two Nilphamari sub-districts. It also evaluated community knowledge on STH control and deworming coverage in both areas for all SACs. Saidpur (intervention) and Kishoregonj (control) sub-districts, in Nilphamari, were surveyed in December 2019. The survey included SACs and their parents. Among SGCs, the intervention group (89.0%) had higher deworming coverage than the control group (75.5%). In the intervention group, 59.9% of OSCs received the deworming tablet versus 24.6% in the control group. Community involvement activities including door-to-door visits, courtyard gatherings, and miking benefited both SACs and their primary caregivers. SACs living in the intervention region, awareness of the last pill distribution date, and caregivers observing BRAC workers in action, were linked to SAC deworming coverage. Re-strategizing the deworming programme to include the OSCs is vital and suggests timely action. Building community awareness and periodic epidemiological assessment can further facilitate an improved drug intake.

## 1. Introduction

Soil-transmitted helminth (STH) infections affected more than 1.5 billion people worldwide in 2020, most of whom reside in the impoverished parts of Asia and Africa. Additionally, STH-related infection was responsible for the loss of approximately 1.9 million disability-adjusted life-years (DALYs) in 2017 [1]. School-aged children (SAC) are among the most vulnerable populations to this group of diseases [2]. Ensuring access to preventive chemotherapy or deworming tablets is the recommended and most sensible public health action among the SACs [3,4].

In Bangladesh, STH infection is endemic in all 64 districts. About one-third of the general population and two-fifths of SACs are in danger of being infected in rural areas [5]. To fight the burden, the Bangladeshi government has been focusing its resources on a biannual school-based deworming pill distribution programme since 2008 [6]. So far, the programme has managed to arrange 23 rounds of deworming sessions up to January 2020 [7].

Because of school-based deworming, the STH infection rate has decreased notably in Bangladesh. The reported national deworming coverage is close to one hundred per cent [8]. Dhakal et al. reported a decreased infection rate among all risk groups in ten endemic districts [7]. However, the programme could not reach every child needing deworming pills, specifically out-of-school children (OSCs) [9].

In Bangladesh, about 3% of children from the primary level and 31% of children from the upper secondary level drop out of school [10]. This is often due to their low-income families, poor living conditions, as well as residence in hard-to-reach areas [11,12]. Additionally, some may remain enrolled in informal or unregistered educational institutions [9]. In some cases, encouraging them to attend the deworming sessions through their school-going peers was effective [13]. However, the deworming coverage in those setting was reported as persistently low [14].

STH infection can reduce a child’s future productivity, halting economic growth in a developing country such as Bangladesh [15]. Bangladesh’s school-based deworming programme should reach all school-aged children regardless of attendance. Recognizing the need, BRAC, a well-known international NGO, implemented a comprehensive STH control project in Saidpur sub-district, Nilphamari, Bangladesh. The project aimed to identify and treat OSCs in Saidpur and educate all risk groups in the area about STH infection and prevention. In 2018, around 25% of school-aged children in Saidpur did not attend school [16].

Reaching all school-aged children is vital for achieving STH control. It is also crucial to monitor the progress of the programme by conducting surveys among the risk groups, because the institutionally reported coverage data can give the wrong impression from time to time [14]. There has been a notable gap reported for deworming coverage among the school-going children (SGCs) and OSCs [3,12].

Henceforth, this study aimed to assess and compare the deworming coverage among SGCs and OSCs in two sub-districts of Nilphamari, Bangladesh. The aim was to assess differences in deworming coverage in two subdistricts with and without the BRAC’s deworming intervention. We also identified the community knowledge regarding STH control and the factors associated with deworming coverage in the two areas for all SACs.

## 2. Materials and Methods

### 2.1. Study Design

The post-test only with the control study is part of a larger study that relied on an embedded mixed-method design. In this design, one dataset provides a supportive secondary role in a study based primarily on the other data type [17]. We have focused only on the quantitative component in this paper.

### 2.2. Study Site and Target Population

The data collection took place in December 2019 in Saidpur and Kishoregonj, two sub-districts of Nilphamari district, Bangladesh. Saidpur was selected as the intervention site and Kishoregonj was selected as the control site. This was deemed appropriate as the sub-districts are neighbours with a similar population size (according to the national census of 2011, Saidpur has a population of 264,461 inhabitants, and Kishoregonj has a population of 261,069 inhabitants) and the government’s school-based deworming programme was running in both places. BRAC ran their deworming programme in Saidpur for three years. Our target population was SACs aged 5–16 years (both school-going and out-of-school) and their primary caregivers.

### 2.3. Sampling Strategy

**Figure 1:**
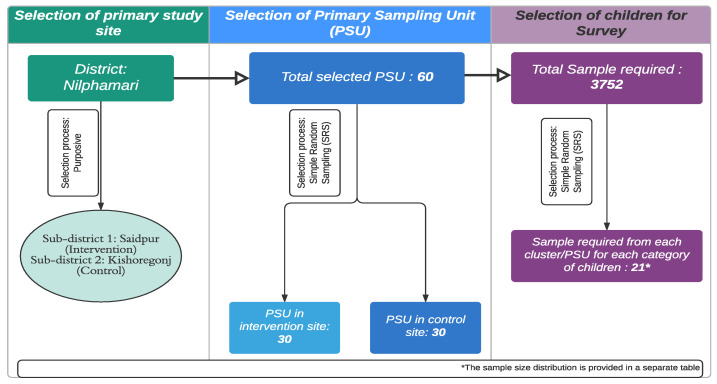
Sampling procedure followed for the selection of samples.

The survey used a two-stage cluster sampling procedure with villages/wards as the primary sampling units (PSU). Figure 1 shows the sampling technique. A home listing was utilized as the sampling frame to select the desired number of children from each PSU chosen randomly. BRAC provided the intervention site household list, whereas the researchers developed the control site household list.

### 2.4. Sample Size Calculation

The sample size for the survey was calculated using the following formula with finite population correction.

(1) $$n=\frac{NZ^{2}P\left(1-P\right)}{d^{2}\left(N-1\right)+Z^{2}P\left(1-P\right)}$$

where *n* = sample size, *Z* = *Z* statistic for a level of confidence, *N* = population size, *P* = expected prevalence, and *d* = precision (in a proportion of one). One cross-sectional survey conducted in 100 villages in rural Bangladesh reported a deworming coverage of 52.4% among children who were 5–14 years old [5]. This coverage was considered the ‘*P*’ for the sample size calculation for this research.

Each study site’s sample size was computed independently for the SGCs (5–11 years and 12–16 years) and the OSCs (5–16 years). The determined sample size was 379 SGCs for ages 5–11 and 377 SGCs for ages 12–16 with a 95% level of significance and a 5% precision estimate. A 1.5 percent design effect and a 10% nonresponse rate were also considered. Both the intervention and control targeted the same number of SGCs and OSCs (Table 1).

A few PSUs in the control area did not have the required number of OSCs. Oversampling was performed proportionally from villages with more than 21 OSCs to cover for the gap. The calculation steps are detailed in Appendix A.

### 2.5. Data Collection and Analysis

A systematic questionnaire was used to survey the groups. Before collecting data, the survey instrument was pretested and tweaked as needed. Thirty data collectors conducted face-to-face interviews after receiving rigorous training. Data gathering was conducted using ODKCollect on tablet computers. Stata^®^ 15 (software by StataCorp LLC, College Station, TX, USA) was used to analyse the data, and the statistical software ‘R’ was used to prepare some figures. Data were described using descriptive statistics and cross-tabulation. The outcome variable (SACs deworming coverage status) was correlated with the other variables using multivariate logistic regression. The wealth quintal was assessed using the verified equity measurement tool [18].

### 2.6. Ethics Statement and Consent Procedures

The study was approved by the BRAC JPGSPH Institutional Review Board. All survey participants gave informed consent. An assent form was utilized to obtain consent from the caregivers to collect information from the SACs. Participants had the right to withdraw from the study at any time.

## 3. Results

### 3.1. Demographic Information of the Target Children and Their Caregivers

Data acquired from the control area were slightly different (Table 2). The intervention area’s SGCs were primarily female (53.6%), while the OSCs were male (63.9%). Over 85.0% of the primary caregivers for all SACs were female and their biological parents.

### 3.2. Self-Reported Deworming Coverage among the SACs

**Figure 2:**
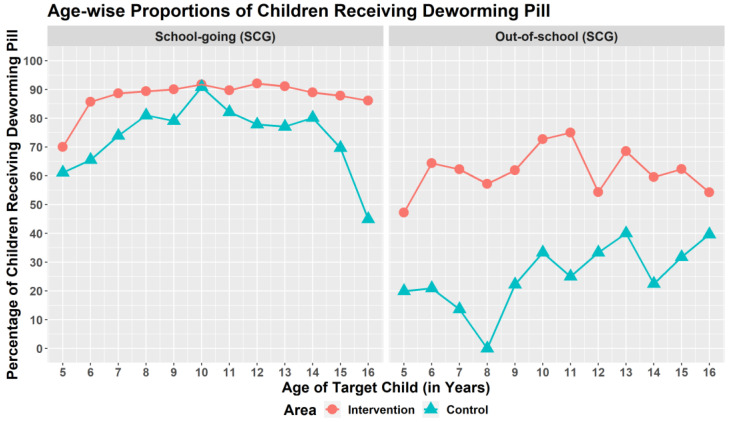
Age-wise proportions of children receiving deworming pill.

Overall, 2559 SACs (69.2%) received deworming medicine. The pill was given to 79.4% of SACs in the intervention site and 59% of SACs in the control site. In both areas, SGCs had higher deworming coverage than OSCs (Figure 2).

### 3.3. Knowledge Assessment of the SACs and Their Primary Caregivers

In total, 78.2% of the SGCs in the intervention area reported knowing the necessity of taking the deworming pill, while 61.0% of the OSCs reported the same. On the other hand, both SGCs and OSCs from the control site were marginally less knowledgeable about the necessity (77.4% and 57.5%, respectively) of taking deworming tablets than the participants from the intervention area. Comparatively, fewer SGCs and OSCs knew about the symptoms related to STH infection in both areas (Table 3).

More than 90 percent of caregivers knew about the importance of their child taking the deworming pill in the intervention area (97.1% for SGCs and 90.7% for OSCs). In the intervention area, 66.8% of SGC caregivers and 56.2% of OSC caregivers knew that the deworming campaign happens twice a year. However, only 24.4% and 19.6%, respectively, could recall the exact time of the last deworming campaign.

Similarly, 45.3% of SGC caregivers and 34.3% of OSC caregivers in the control area knew the deworming campaign happens twice a year. Nonetheless, only 8.4% and 2.0% of the caregivers from the control area could recall the exact time of the last deworming campaign in their area.

### 3.4. Assessment of Caregiver’s Knowledge about Deworming Project Activities

Most caregivers in the intervention area were informed of the necessity of taking the deworming pill by the community workers from BRAC (49.0% among SCGs and 57.1% among OSCs). In the control area, caregivers of the SGCs mentioned their child’s teachers as the source of information (22.8%), while OSC caregivers were more likely to mention the BRAC community workers. Additionally, more caregivers in the intervention area (33.7% and 35.6% of caregivers for SGCs and OSCs, respectively) saw BRAC community workers going door-to-door to raise deworming awareness, compared to those in the control area (4.1% and 2.7% caregivers for SGCs and OSCs, respectively) (Table 4).

### 3.5. Factors Associated with the Deworming Coverage of the SACs

Children residing in the intervention area had a 1.80 times (CI: 1.45–2.24) higher chance of receiving the deworming pill compared to those residing in the control area. OSCs residing in the intervention area had an 82% lower chance of receiving the pill, while the OSCs residing in the control had an even lower chance (88%).

Similarly, SACs who were aware that “not taking the pill will make them sick” (AOR: 1.59; CI: 1.11–2.28 in the intervention and AOR: 1.78; CI: 1.26–2.50 in the control) were more likely to take the pill (Table 5).

Furthermore, caregivers learning about deworming sessions from BRAC community workers (AOR: 2.07; CI: 1.49–2.88 in the intervention and AOR: 1.65; CI: 1.19–2.28 in the control) and witnessing them conducting community outreach about deworming campaigns (AOR: 1.79; CI: 1.23–2.59 in the intervention and AOR: 1.95; CI: 0.91–4.19 in the control) significantly improved the likelihood of their SACs receiving the treatment (Table 5).

## 4. Discussion

This study revealed that remarkable progress has been achieved in deworming pill distribution to school-aged children. However, despite the advancements found in our investigation, there remain discrepancies in the coverage status of SGCs and OSCs. Additionally, school enrolment status, prior understanding of the medication distribution and its relevance, and caregiver expertise with community mobilization all influenced the drug coverage among SACs.

The coverage for SGCs in the intervention area exceeded the WHO target (≥75% coverage among all risk groups) for eliminating STH infection by 2030 compared to children living in the control area. However, the coverage in the intervention area for the OSCs was poor, and it was poorer still in the control area. Other authors reported similar findings from Bangladesh [9,12,16] and Ethiopia [14]. Given that SGCs are frequently the primary target of deworming programs, and there is a lack of protocol to integrate the OSCs [9], it is unsurprising that coverage is higher among the SGCs. Furthermore, OSCs often drop out of school to work, so focusing on exclusively a school-based programme may not be enough. The WHO advises targeting specific risk groups with STH-infection information [13]. Educating through community workers and child peer groups can help bring all SGCs and OSCs under the deworming umbrella. The latter has previously benefited Ethiopia [13].

On the other hand, the reported coverage for SGCs and OSCs was significantly lower than the nationally reported coverage [8]. Previous research has found that reporting by pill distributors can sometimes result in over-reporting [9,14]. Because few informal educational institutions are listed in the local registry, those children are often excluded from school-based deworming [9]. Therefore, it is critical to initiate alternative strategies to access those children.

The children and their caregivers in both areas were aware of the need to take deworming tablets and the consequences of not taking them. However, there was a gap between knowledge and action regarding community mobilization. Two-thirds of respondents in the control region were unaware of the deworming pill distribution authority. Knowledge of medication administration often influences coverage on such a massive scale [19,20].

This study also showed that the chances of children being covered increased significantly if both children and their caregiver(s) knew the exact date of the last pill distribution. If too many youngsters were unaware of the deworming session, they were more likely to miss the pill distribution, resulting in higher expenses for future deworming camps [21]. Incorporating deworming material into the school curriculum can also help youngsters understand the necessity [22,23,24].

Parents and other vital caregivers can significantly influence their children’s lifestyle choices by advocating a healthy lifestyle [25]. Community sensitization is the key to disseminating essential information. The coverage in the intervention areas where caregivers were most exposed to community sensitization activities was significantly better. The findings suggest that community mobilization is critical for a successful deworming program. A community-based organization, such as BRAC, can facilitate information dissemination to caregivers and increase coverage. Studies also reported improved health outcomes when deworming was combined with other popular health programs such as immunization camps and malaria eradication [26,27,28]. Integration with community mobilization activities of immunization or other health programs can strengthen the deworming program.

Even if caregivers were aware that their child needed the deworming tablet, failing to remember the date may have lessened the impact of prior knowledge. The implementation of the STH control programme in Bangladesh was hampered by a lack of communication and information among stakeholders [9]. Similar gaps impede programme acceptability within the community [29]. Ensuring that healthcare professionals who disseminate information receive additional communication training will assist them in developing stronger relationships with communities.

Some limitations of this research should be noted. Recall bias may play a part when remembering drug uptake history. Furthermore, this cross-sectional research could not establish any cause–effect relationships.

## 5. Conclusions

Our study provided evidence that deworming coverage among OSCs was much lower than among SGCs. Knowledge about different aspects of the STH control programme was higher in the area with BRAC intervention than the control, and knowledge was better among the adult caregivers than the SACs. Awareness about the last deworming pill distribution timing and community mobilization activities were important factors associated with children’s deworming coverage. Continuous assessment of deworming coverage using periodic epidemiological surveys in other endemic areas among different risk groups is essential to better understanding the drug uptake pattern. Engaging community-based organizations, such as BRAC, and a well-coordinated community mobilization strategy with other health programs are required to bring out the full potency of the STH control program.

## Figures and Tables

**Table 1:** Calculated Sample size for the survey.

	OSCs		SGCs			
Selected Study Sites	Saidpur (Intervention)	Kishoregonj (Control)	Saidpur (Intervention)		Kishoregonj (Control)	
Age Strata	5–16 years		5–11 years and 12–16 years			
Number of PSUs	30					
Required Sample Size/Age Group	617	617	631	628	631	628
Interviews Conducted	613	598	617	623	623	623

**Table 2:** Demographic information and self-reported deworming coverage of OSCs.

	Responses			
Variables	Intervention (N = 1853)		Control (N = 1844)	
	SGC (*n* = 1240)	OSC (*n* = 613)	SGC (*n* = 1246)	OSC (*n* = 598)
Mean Age of the Target Children				
Mean Age, in years (±SD)	11 (3.1)	11 (4.1)	11 (3.1)	9 (4.4)
Gender of targeted SAC				
Female	664 (53.6)	221 (36.1)	665 (53.4)	220 (36.8)
Male	576 (46.5)	392 (63.9)	581 (46.6)	378 (63.2)
Mean Age Primary Caregivers *				
Mean Age, in years (±SD)	35 (8.2)	36 (9.4)	36 (9.2)	35 (9.9)
Gender Primary Caregivers *				
Female	1042 (88.2)	510 (89.3)	1047 (87.8)	485 (86.30
Male	139 (11.8)	61 (10.7)	146 (12.2)	77 (13.7)
Relationship of the OSCs to their Primary Caregivers *				
Parents	1088 (92.1)	505 (88.4)	1060 (88.9)	490 (87.2)
Other Caregivers	93 (7.9)	66 (11.6)	133 (11.1)	72 (12.8)
Wealth status ^#^				
Poorest	7 (0.6)	17 (3.0)	29 (2.4)	23 (4.1)
Poor	118 (10.0)	100 (17.5)	471 (39.5)	264 (47.0)
Middle	195 (16.5)	122 (21.4)	343 (28.8)	166 (29.5)
Rich	512 (43.4)	236 (41.3)	296 (24.8)	88 (15.7)
Richest	349 (29.6)	96 (16.8)	54 (4.5)	21 (3.7)

Note: *n* (%) is reported unless otherwise specified; asterisk (*) indicates that some of the OSCs were from the same HHs, so primary caregivers’ information is given once; hash (^#^) indicates the asset index of one OSC was not available.

**Table 3:** Distribution of SACs and their caregiver’s knowledge across intervention and control groups.

	SACs (N = 3697)				Caregivers (N = 3507)			
Variables	Intervention		Control		Intervention		Control	
	SGC, *n* = 1240	OSC, *n* = 613	SGC, *n* = 1246	OSC, *n* = 598	SGC, *n* = 1181	OSC, *n* = 571	SGC, *n* = 1193	OSC, *n* = 562
Importance and reason for taking the deworming pill								
Taking the deworming pill is necessary	970 (78.2)	374 (61.0)	964 (77.4)	344 (57.5)	1147 (97.1)	518 (90.7)	1088 (91.2)	481 (85.6)
Taking the deworming pill will keep them from getting the worm	916 (73.9)	330 (53.8)	843 (67.7)	291 (48.7)	1120 (94.8)	507 (88.8)	1023 (85.8)	446 (79.4)
Not taking the pill will make them feel sick	985 (79.4)	374 (61.0)	947 (76.0)	331 (55.4)	1102 (93.3)	510 (89.3)	1067 (89.4)	485 (86.3)
Knowledge about STH infection-related symptoms								
Knows bloating is a symptom	606 (48.9)	236 (38.5)	573 (46.0)	221 (37.0)	824 (69.8)	386 (67.6)	848 (71.1)	380 (67.6)
Knows vomiting is a symptom	449 (36.2)	173 (28.2)	361 (29.0)	130 (21.7)	649 (55.0)	302 (52.9)	550 (46.1)	235 (41.8)
Knows lack of appetite is a symptom	178 (14.4)	66 (10.8)	155 (12.4)	50 (8.4)	285 (24.1)	120 (21.0)	231 (19.4)	95 (16.9)
Knows indigestion is a symptom	356 (28.7)	144 (23.5)	303 (24.3)	110 (18.4)	576 (48.8)	299 (52.4)	534 (44.8)	204 (36.3)
Knows spitting frequently is a symptom	364 (29.4)	133 (21.7)	384 (30.8)	141 (23.6)	529 (44.8)	251 (44.0)	562 (47.1)	230 (40.9)
Knows itching in the anus is a symptom	149 (12)	60 (9.8)	194 (15.6)	80 (13.4)	300 (25.4)	125 (21.9)	356 (29.8)	157 (27.9)
Knows the type of illness caused by STH infection								
Knows it causes malnutrition	330 (26.6)	125 (20.4)	296 (23.8)	132 (22.1)	551 (46.7)	235 (41.2)	489 (41)	215 (38.3)
Knows it causes anaemia	135 (10.9)	52 (8.5)	148 (11.9)	57 (9.5)	235 (19.9)	95 (16.6)	224 (18.8)	95 (16.9)
Knows it causes indigestion	236 (19.0)	98 (16.0)	208 (16.7)	70 (11.7)	325 (27.5)	137 (24.0)	305 (25.6)	141 (25.1)
Knows it causes diarrhoea	269 (21.7)	106 (17.3)	270 (21.7)	111 (18.6)	399 (33.8)	170 (29.8)	387 (32.4)	163 (29.0)
Knows the pathways of STH transmission								
Eating with dirty hands	633 (51.0)	224 (36.5)	555 (44.5)	200 (33.4)	810 (68.6)	393 (68.8)	758 (63.5)	318 (56.6)
Not washing hands after defecation	570 (46.0)	184 (30.0)	465 (37.3)	156 (26.1)	740 (62.7)	336 (58.8)	653 (54.7)	288 (51.2)
Not washing hands before eating food	319 (25.7)	111 (18.1)	263 (21.1)	85 (14.2)	424 (35.9)	176 (30.8)	389 (32.6)	148 (26.3)
Drinking contaminated water	93 (7.5)	28 (4.6)	75 (6.0)	21 (3.5)	122 (10.3)	42 (7.4)	92 (7.7)	37 (6.6)
Not wearing shoes	541 (43.6)	178 (29.0)	480 (38.5)	178 (29.8)	698 (59.1)	304 (53.2)	631 (52.9)	284 (50.5)
Open defecation	50 (4.0)	22 (3.6)	79 (6.3)	20 (3.3)	75 (6.4)	38 (6.7)	122 (10.2)	54 (9.6)

Note: *n* (%) is reported unless otherwise specified.

**Table 4:** Caregiver’s knowledge about deworming project activities.

Variables	Intervention, SCG	Intervention, OSC	Control, SCG	Control, OSC
	*n* = 1181	*n* = 571	*n* = 1193	*n* = 562
Sources that informed caregivers about the importance of the deworming pill				
Community worker from BRAC	579 (49.0)	326 (57.1)	228 (19.1)	107 (19.0)
Teacher of their child	313 (26.5)	31 (5.4)	272 (22.8)	49 (8.7)
Medical Doctors	123 (10.4)	60 (10.5)	137 (11.5)	73 (13.0)
Through Visual aids/Miking	91 (7.7)	30 (5.3)	83 (7.0)	27 (4.8)
Types of sensitization activity seen before the deworming campaign in the area				
Miking	402 (34.0)	197 (34.5)	65 (5.4)	21 (3.7)
Household visit by community workers	278 (23.5)	161 (28.2)	29 (2.4)	14 (2.5)
Courtyard meeting with women	152 (12.9)	78 (13.7)	25 (2.1)	11 (2.0)
Courtyard meeting with adolescent girls	16 (1.4)	8 (1.4)	-	-
Courtyard/Outdoor meeting with men	23 (1.9)	4 (0.7)	-	1 (0.2)
In-school programs with children	38 (3.2)	4 (0.7)	7 (0.6)	-
Cable TV message	11 (0.9)	4 (0.7)	5 (0.4)	2 (0.4)
Knowledge about the workers seen engaged in household visits in the area				
Community worker from BRAC	398 (33.7)	203 (35.6)	49 (4.1)	15 (2.7)
Community worker from government	145 (12.3)	49 (8.6)	71 (6.0)	23 (4.1)

Note: *n* (%) is reported unless otherwise specified.

**Table 5:** Factors associated with deworming coverage of the SACs.

Variables	Intervention		Control	
	Adjusted Odds Ratio or, AOR (95% Confidence Interval)	*p*-Value	Adjusted Odds Ratio or, AOR (95% Confidence Interval)	*p*-Value
Child not going to school	0.18 (0.14–0.25)	0.000 ***	0.12 (0.09–0.15)	0.000 ***
Child’s gender is female	1.24 (0.93–1.64)	0.139	0.91 (0.72–1.16)	0.447
Caregiver’s gender is female	1.07 (0.70–1.64)	0.743	1.37 (0.97–1.94)	0.073 *
Household belongs to 2nd/poor wealth quintal	0.82 (0.28–2.37)	0.714	0.95 (0.47–1.93)	0.895
Household belongs to 3rd/middle wealth quintal	0.67 (0.24–1.90)	0.453	1.12 (0.55–2.30)	0.751
Household belongs to 4th/rich wealth quintal	0.75 (0.27–2.08)	0.579	0.90 (0.43–1.86)	0.77
Household belongs to 5th/richest wealth quintal	0.84 (0.29–2.42)	0.751	0.96 (0.39–2.37)	0.923
The child knows taking the deworming pill is important	0.92 (0.53–1.59)	0.77	1.89 (1.21–2.96)	0.005 ***
Child knows taking the deworming pill will prevent them from harm	1.62 (0.92–2.87)	0.095 *	0.64 (0.40–1.02)	0.059 *
Child knows not taking the pill will make them fall sick	1.59 (1.11–2.28)	0.011 **	1.78 (1.26–2.50)	0.001 ***
Child knows precise date of the last pill distribution	2.49 (0.92–6.75)	0.074 *	1.68 (0.90–3.16)	0.106
Caregiver knows taking the deworming pill is important	0.99 (0.40–2.46)	0.987	1.56 (0.91–2.69)	0.107
Caregiver knows taking the deworming pill will prevent harm for their child	0.67 (0.39–1.12)	0.127	1.51 (1.00–2.27)	0.048 **
Caregiver knows not taking the pill will make the child fall sick	4.63 (2.32–9.25)	0.000 ***	5.68 (2.48–12.99)	0.000 ***
Caregiver knows the precise date of last pill distribution	2.57 (1.53–4.32)	0.000 ***	4.12 (1.53–11.13)	0.005 ***
Caregivers learned about deworming sessions from BRAC community workers	2.07 (1.49–2.88)	0.000 ***	1.65 (1.19–2.28)	0.003 ***
Caregivers learned about deworming sessions from visual aid/miking	1.37 (0.73–2.56)	0.323	0.93 (0.57–1.52)	0.782
Caregivers saw sensitization activity-courtyard meeting with women	0.80 (0.49–1.32)	0.382	1.58 (0.57–4.38)	0.383
Caregivers saw sensitization activity-miking	1.33 (0.92–1.92)	0.131	2.77 (1.41–5.44)	0.003 ***
Caregivers saw sensitization activity-household visit by community workers	1.09 (0.72–1.65)	0.697	0.24 (0.11–0.53)	0.000 ***
Caregivers saw community workers from the government going door-to-door	2.42 (1.37–4.25)	0.002 ***	2.08 (1.14–3.79)	0.017 **
Caregivers saw community workers from BRAC going door-to-door	1.79 (1.23–2.59)	0.002 ***	1.95 (0.91–4.19)	0.085 *

*** *p* < 0.01, ** *p* < 0.05, * *p* < 0.1.

## Data Availability

The data presented in this study are available on request from the corresponding author. The data are not publicly available due to their identifiable nature.

## Appendix A

Steps followed for determining the sample size for each village where the desired number of OSC were not available.

Step 1: We first calculated the deficit of OSC from villages with fewer than 21 OSC and the excess of OSC from villages with more than 21 OSC using the following formulae:

OSC deficit=21−# of OSC found

# of excess OSC=# of OSC found−21

Step 2: We calculated the total deficit of OSC from villages with fewer than 21 OSC:

$$Total\;OSC\;deficit=\sum^{\;}OSC\;deficit,$$

where the summation was only for those villages that had fewer than 21 OSC.

We calculated the number of excess OSC in those villages where more than 21 OSC were found:

$$Total\;\#\;of\;excess\;OSC=\sum^{\;}\#\;of\;excess\;OSC,$$

where the summation was only for those villages with more than 21 OSC.

Step 3: We calculated the number of excess OSC to be sampled from each of those villages with more than 21 OSC, in proportion to the number of excess OSC found in that village:

$$\#\;of\;excess\;OSC\;sampled=\frac{\#\;of\;excess\;OSC}{Total\;\#\;of\;excess\;OSC}\times Total\;OSC\;deficit$$

Step 4: Finally, we calculated all the OSC from villages with fewer than 21 OSC found, and we added up the desired number of 21 with the number of excess OSC to be sampled from villages with more than 21 OSC. The final sample size for OSC from each of the selected villages in Kishoregonj was calculated as:

# of OSC sampled=# of OSC found if # of OSC found<21

# of OSC sampled=21+# of excess OSC sampled if # of OSC found≥21

Following the above procedure, we obtained 628 OSC in total from Kishoregonj, against the desired number of 630.
